# Enlisting Probiotics to Combat Recurrent Urinary Tract Infections in Women—A Military Strategy for Meeting the Challenge

**DOI:** 10.3390/antibiotics12010167

**Published:** 2023-01-13

**Authors:** Richard A. Watson

**Affiliations:** Hackensack Meridian School of Medicine, Nutley, NJ 07110, USA; richard.watson@hmhn.org

**Keywords:** urinary tract infections, probiotics, microbiome

## Abstract

For decades, the potential role of probiotics in the prevention and treatment of recurrent urinary tract infections has been extensively studied. However, achieving an effective problem-solving strategy has thus far proven elusive. Perhaps adopting a military paradigm might expedite our assault on chronic, recurring bacteriuria in women. What is needed is a targeted strategy with specific attention to (1) the enemy: the case-specific uropathogen; (2) the battlefield: the extraordinarily complex interplay of factors within the bladder, unique to a given patient, which interface with profoundly important influences from the gut biome, as well as the vaginal biota; (3) the weapon: an antimicrobial probiotic with demonstrated activity against that specific uropathogen; (4) a new strategy: taking these complexities into account, we posit a key role for the instillation of case-specific lactobacilli directly into the bladder of the designated patient. This newly proposed, targeted intervention might be termed “Probiotic Intravesical Organic Therapy—PIVOT”; and (5) the long campaign: reaching clinically proven success may entail a long campaign. However, already, on many fronts, the elements necessary for victory recently seem to be falling into place.

## 1. Introduction

For decades, the potential role for probiotics in the prevention of recurrent urinary tract infections in women (rUTI) has been extensively studied. However, achieving an effective problem-solving strategy has thus far proven elusive. Many practitioners still follow the therapeutic merry-go-round of treating these infections with repeated courses of antibiotics, which only result in the further recurrence of infections, frequently caused by progressively resistant organisms. However, major new breakthroughs in our understanding of the very nature of rUTIs now offer the opportunity for a radical change in our assessment, treatment and prevention of this condition. Genomic analyses revolutionize traditional concepts, introducing novel terminology and radical new insights [1]. Normal, healthy urine is *not* sterile [2]. Bladder health is not dependent upon the presence or absence of any single uropathogen, but rather upon the interplay of a balanced ‘medley’ of organisms within the urobiota of the bladder [3,4]. The gut microbiome—the healthy composition and interplay of bacteria within the bowel—has a major influence on bladder health [3,4]. Oral probiotics have long been used to prevent rUTIs, but with mixed results [5]. Genomic research here, too, promises to radically upgrade this intervention [1]. Strains of lactobacilli have now been identified that have proven antibacterial activity against a specific uropathogen [6,7]. There is the potential for bioengineering ‘designer probiotics’ for even better targeted use [8]. Additionally, preliminary clinical research has demonstrated the feasibility of introducing these lactobacilli directly into the bladder [9,10,11].

As a retired Army urologist, it occurs to me that perhaps adopting a military paradigm might help us to accommodate this sea change in concepts and expedite a preemptive assault on rUTIs in women. Let us look at a targeted strategy with specific attention to (1) the enemy, (2) the weapon, (3) the battlefield and (4) the long campaign to victory.

(1) The enemy: Gram-negative bacteria resistant to antibiotics and innate immune defenses: Multitudinous Gram-negative bacteria have been implicated. It is widely accepted that one or more of these uropathogens are harbored in the bowel and vagina of susceptible patients. 

Antibiotics, when given to eradicate one infection, predispose to the development of another. Antibiotics eliminate disease-causing bacteria from the bladder, but not necessarily from the intestines. Subsequently, now-resistant bacteria in the gut can multiply and spread back into the bladder once again. 

Antibiotics do not prevent future infections, nor do they permanently clear rUTI-causing strains from the gut. They may even make recurrence more likely by keeping the microbiome in a disrupted state. At the same time, repeat cycles of antibiotics wreak havoc on the community of healthy bacteria that normally live in the intestines—the normal gut microbiome. Beneficial commensal bacteria, important to maintaining bladder health, may fall victim to friendly fire [12]. Researchers have found that, for women with rUTIs, a round of antibiotics usually does clear up the symptoms, but the relief is only temporary. A quarter of woman go on to develop a second rUTI within 6 months [13].

(2) The battlefield: Treat the condition in the affected bladder: (Remaining mindful of the extraordinarily complex interplay of factors within the bladder. These factors are unique to each patient. They interface with and are profoundly influenced by the gut biome, as well as the vaginal biota).

A joint study from Washington and Harvard Universities [12] indicates that maintaining a healthy gut microbiome, more importantly than eradicating pathogenic *E. coli*, is central to avoiding rUTI. Their study compared the findings in women who had rUTIs with those who did not. Surprisingly, these researchers found that the difference between healthy and cystitis-prone women was not a result of the kind of *E. coli* in the intestines or even the presence of *E. coli* in their bladders. Both groups carried *E. coli* strains in the gut capable of causing rUTIs, and those strains occasionally spread to the bladders in both cases [12]. The difference was in the make-up of the gut microbiome. Patients with repeated infections showed a decreased diversity of healthy gut microbiota, which allowed more opportunities for disease-causing species to gain a foothold in the bladder and multiply there. Notably, the microbiome of women with rUTIs was scarce in bacteria that produce butyrate, a short-chain fatty acid with anti-inflammatory effects [13]. 

Moreover, women with rUTIs were unable to clear bacteria from their bladders before they caused disease because of their bladder’s lack of a healthy immune response. This response is seemingly mediated by the gut microbiome, which normally provides defense against pathogenic bacterial invasion of the bladder. Maintenance of a healthy gut biome is essential to bladder hygiene. The function of a healthy microbiome is not dependent alone upon its bacterial composition. Rather, it requires the orchestration of multiple, complex, dynamic bacterial interactions—a symbiotic ‘repertoire’. Disruption of this interplay results in ‘dysbiosis’ [3,4,12].

Attempts to restore a healthy gut microbiome have entailed the use of probiotic foods, investigational vaccines, and experimental mannoside drugs [12]. Enteral probiotics have long been used in an attempt at a body-wide transformation, eradicating uropathogens throughout the gut and vagina. Benefit to the bladder would be indirect.

The vagina, with healthy microbiota, might be seen as a fortress protecting the vulnerable bladder. Conversely, bacterial dysbiosis may convert the vagina into a launching pad for bladder invaders. Vaginal microbiome transplants have been advocated to restore its integrity [14]. Fecal transplants, designed to restore a healthy gut microbiome, have moved further into the realm of mainstream therapy, since the US FDA recently gave its first-ever approval to fecal transplant therapy [15].

Pioneering metagenomic investigations into the urinary tract microbiome have now opened up a new world of understanding regarding the pathophysiology of rUTIs. Recent data characterizing the human urinary microbiota, in health and disease, defy the decades-old clinical practice of striving for urinary tract sterility in urogenital health [16,17,18]. Counter to widely-held clinical belief, healthy urine is not sterile. Sophisticated assessment of the urobiome, using Next-Generation Sequencing (NGS) analyses, confirmed via metaproteomics, challenges this traditional concept [16]. The healthy urine microbiome is characterized by a preponderance of *Lactobacillus* in women and corynebacterium in men. The top ten bacterial taxa were Lactobacillales, Enterobacteriales, Actinomycetales, Bacillales, Clostridiales, Bacteroidales, Burkholderiales, Pseudomonadales, Bifidobacteriales, and Coriobacteriales [17].

Moreover, these researchers found that: (1) when collected by the routine method of “clean, midstream catch”, healthy urine is not aseptic; (2) microbiomes in healthy versus neuropathic bladder urine differed by patients’ gender; (3) the urine microbiome of patients with neuropathic bladders, but without symptomatic cystitis, also differed from that of healthy controls; and (4) the microbiome of self-catheterizing people with neuropathic bladders varied, depending on duration of exposure to and type of urinary catheter.

Findings suggest that lactobacilli may be an important commensal organism commonly present during states of health, more so in females than in males, and that, conversely, the microbiomes of at-risk populations may be characterized by a distinct lack of *Lactobacillus*. Its absence may afford an environment more conducive to the growth of pathogenic microorganisms. Together, these findings suggest that the clinical objective of ‘sterile’, microbe-free urine may not be advantageous, or even long achievable [17]. Indeed, metagenomics research currently suggests that the presence of a stable microbiome is essential to maintaining bladder health. There needs to be a normal urinary tract bacterial repertoire [3,4]. Recent culturomics research has isolated a total of 450 diverse bacteria species within the urine—including 256 not previously found in the urine and 18 completely new species [4]. More important than the presence or absence of specific bacteria may be the perturbation of harmony within the urinary microbiota. This “urinary tract dysbiosis” may precede development of a clinical infection. 

There is a dynamic ongoing interplay between the microbiota of the bladder, vagina, and bowel. Meštrović et al. note that “The gut, vagina and urinary bladder represent a trifecta of anatomical sites jointly implicated in the pathogenesis of rUTI, with resident microbiota either serving as a potential reservoir of uropathogenic bacteria, or protecting us from their potential to cause UTI” [18].

This genomic age may presage, beyond a change in the treatment and prevention paradigm, a revolution of our understanding of rUTIs. Johns Hopkins authority Prof. Thomas Finucane asserts that “Everyone is bacteriuric. From this perspective, most people who are treated for a “UTI” would probably be better off without treatment…Mindful decisions about antibiotic use will require a far better understanding of how pathogenicity arises within microbial communities…Emphasizing potential harms and uncertain benefits, would reduce overtreatment. Emphasizing the microbiome’s significance and using the term “urinary tract dysbiosis,” instead of “UTI,” might also help and might encourage mindful study of the relationships among host, aging, microbiome, disease, and antibiotic treatment” [2].

(3) The weapon: A specific *Lactobacillus* with proven antimicrobial activity against the case-specific causative organism.

Rather than a “buckshot” approach, using a random mix of probiotic bacteria, we would utilize advanced technology to identify a specific *Lactobacillus* with proven activity against the identified, case-specific uropathogen.

Current research has found that different lactobacilli demonstrate varying antibacterial activity. Studies at the University of Alabama concluded that inhibition varied widely among species and strains of urinary lactobacilli. They found a high level of species and strain diversity that warrants future detailed investigations [6].

A recent report in this journal [19] found that lactic acid bacteria, including *Lactobacillus*, *Bifidobacterium*, *Streptococcus*, *Lactococcus*, and *Leuconostoc*, are the predominant groups of bacteria with proven probiotic action. *Lactobacillus* assumes the greatest relevance. This group of bacteria can grow in different habitats using diverse sources of carbon. Via glucose metabolism, these lactic acid bacteria are capable of producing several other metabolites besides lactic acid, such as ethanol and acetic acid. These substances, together with other secondary metabolites, such as organic acids, exopolysaccharides, biosurfactants, enzymes, and bacteriocins, provide a physiologically restrictive environment (e.g., low pH, redox potential, hydrogen sulfide, and peroxide production), making it less suitable for competitors. Bacteriocins are a particular class of exometabolites produced by probiotics that substantially inhibit the competing growth of uropathogens [16].

Various strains of *Lactobacillus* exhibit characteristics that might facilitate targeted antimicrobial activity via cell adhesins and biosurfactants. However, this activity varies substantially from one strain to the next [20]. The antibacterial effect of *Lactobacillus* has been demonstrated against *Clostridium difficile*, *Escherichia coli*, *Shigella* spp., *Streptococcus mutans*, *Pseudomonas aeruginosa*, *Staphylococcus aureus*, and recently against Carbapenemase-Producing Enterobacteriaceae [8]. Research would need to determine which uropathogens (e.g., specific Gram-negative species, such as *E. coli*, *Klebsiella*, or *Pseudomonas*) may be susceptible to probiotic intravesical therapy.

Beyond their direct bacteriocidal activity, intravesical probiotics may have an enduring effect on the internal environment of the bladder. They may modify the urobiome and induce protective changes within the bladder mucosa. Targeted, personalized, intravesical probiotic therapy may not only have efficacy in the immediate treatment of an ongoing infection, but also provide long-term advantages in the promotion of a healthier intravesical environment with reduced risk of subsequent infections.

“Cura personalis”—personalized, precision medicine—calls for tailoring the treatment of a specific patient, based on that patient’s unique genetic and physiologic characteristics [21]. This personalized therapy would target probiotic treatment against a specific bacterial organism with proven susceptibility. We would then be able to test the effectiveness of targeted probiotic therapy, based on the response within this specific, individualized setting.

(4) A new strategy: Treat the condition within the case-specific bladder with targeted probiotic therapy.

Intravesical instillation of lactobacilli with proven activity against the targeted uropathogen in a specific patient stages the locus of treatment directly within the afflicted bladder. Currently, we use intravesical therapy routinely in the treatment of bladder cancers. In addition, intravesical use of antibiotics, analgesics, and steroid preparations are also routinely instilled. Intravesical use of bacteriophages has been advocated for treatment of rUTIs [22]. Even *E. coli* (using a low-virulence strain) have been instilled into the bladder, in order to reduce the recurrence of symptomatic bladder infections in women with incomplete emptying of their bladders [23].

Intravesical instillation of probiotics has proven safe. Lactobacilli do not persist on standard culture following bladder instillation (despite the fact that *Lactobacillus* fragments are detected in NGS screening of urine, even in healthy, asymptomatic patients) [11]. Development of a *Lactobacillus* UTI would be reportable. 

Intravesical instillation of *Lactobacillus*, in particular, has been proven to be safe and well-tolerated in adults and children with neurogenic lower urinary tract dysfunction. In 2019, 103 patients with a neuropathic bladder requiring intermittent self-catheterization underwent a trial of self-instillation of intravesical *Lactobacillus* in response to the development of cloudy or foul-smelling urine. A baseline and a washout period were included in the study. The study achieved its goal of demonstrating the safety, tolerability and practicality of the intervention. However, the outcome in terms of clinical improvement was not sufficient to warrant a therapeutic recommendation for this intervention, pending further study [9,10,11].

In a parallel report of this same first-in-human study, adult and pediatric patients with neuropathic bladders, who needed to self-catheterize periodically, instilled *Lactobacillus rhamnosus* intravesically at the first sign of an impending infection. A total of 96 adults and 7 children were studied. One or more doses of *Lactobacillus* were instilled intravesically in the test group, triggered by cloudy or foul-smelling urine, or the early onset of bladder symptoms. Control patients did not instill probiotics. Patients in the test group demonstrated not only a measureable improvement in bladder symptoms, but also in impeding the development of recurrent prodomal urinary changes, such as cloudy, dark or foul-smelling urine. This finding suggests that early intervention might preclude development of “actionable” bladder infections [11]. A separate proof-of-concept study confirmed that a single intravesical instillation of Lactobacilli is safe in adult and children with neuropathic bladders [12]. These investigators plan further studies to elucidate the role of *Lactobacillus* bladder instillations in this setting.

Failure to show a major improvement may have been due, in part, to several limitations. This study was based on interventions in patients with dysfunctional neuropathic bladders with chronic urinary retention, requiring long-term intermittent self-catheterization. Intervention was limited to one or two instillations at the outset of symptomatic infections. No effort was made to verify any antibacterial activity on the part of the *Lactobacillus* chosen for bladder instillation, let alone to demonstrate its activity against the specific uropathogen cultured in the ongoing infection.

The population was well-suited for this trial in that the patients were already accustomed to self-catheterization on a daily basis, had chronic colonization of their bladder urine, and were at risk of flares of acute cystitis. It remains to be determined how well this experience will translate into the treatment of women with normally functioning bladders who would be undergoing periodic instillations in an outpatient setting.

(5) The long campaign: Development and test-site validation of this concept. 

Victory will not come easily. Capitalizing on transitional research will require several arduous steps.

Not every woman will prove an appropriate candidate. Certainly, women with acutely symptomatic cystitis will continue to be best served by prompt instatement of appropriate antibiotic therapy. The most appropriate candidate for intravesical therapy remains to be determined. Patients with chronic, asymptomatic bacteriuria might be ideal candidates, but does asymptomatic bacteriuria need treatment at all? (Research suggests that untreated bacteriuria may actually reduce the frequency of symptomatic UTIs [24].) For women with recurrent acute UTIs, at what point would intravesical therapy best be applied—During symptom-free intervals? At the onset of a prodome of infection? As an adjunct to definitive antibiotic treatment? 

Field testing this novel application of intravesical therapy will prove expensive in terms of time and effort, involving the sophisticated use of personnel, equipment and complex laboratory investigations. It is unlikely, under current conditions, that medical centers will be able to produce this product within their own facilities. We would need to develop centers that are capable of rapidly identifying an effective strain of *Lactobacillus* and delivering that culture expeditiously to distant treatment centers [20].

There are over 260 species and subspecies of *Lactobacillus* alone [25]. Detecting a probiotic agent with specific antibacterial activity—finding the magic needle in this bacillary haystack—will prove a challenge. 

Once identified, cultures of lactobacilli, or other probiotic strains with proven activity against specific strains of uropathogens, will need to be archived in a bank of cultures, maintained for case-specific correlation and delivery. For each test case, we will next need to verify culture-proven activity of the selected *Lactobacillus* against the case-specific uropathogen, and then quickly prepare that *Lactobacillus* for culture expansion, followed by rapid delivery to the distant clinic site for prompt bladder instillation. Difficulties may arise in upscaling and storing cultures of fastidious lactobacilli and bifiobacterial strains. Freeze-drying or spray-drying may afford prolonged shelf-life stability for storage and transportation at room temperature [24].

Prospective pharmaceutical corporations will question the chances of recouping their investment if the endpoint is merely marketing cultures of a common yogurt byproduct. Could specific lactobacilli or other probiotic strains be patented? While naturally occurring organisms cannot be patented, if the probiotic bacterium is modified to a new strain in the lab, that new strain might be patentable. Additionally, any new methodology, using even a naturally occurring bacterial strain, if it positively impacts health, can be patented [26]. BCG bacilli have been successfully marketed for intravesical instillation. However, lack of profitability has been responsible, in part, for recent shortages worldwide. Dr Sam Chang of Vanderbilt University observes “It is not easy to make BCG; it’s a bacteria that grows slowly, and it has to be kept sterile. There’s no patent on it and little profit in its production and sale” [27].

Developments in “postbiotic” research suggest that use of the metabolic components of lysed probiotic bacilli may achieve significant therapeutic advantage with fewer challenges in terms of production, storage, transportation and delivery [28,29].

An additional advantage to this probiotic approach is that it is natural—‘organic.’ We would be able to avoid the use of antibiotics and other artificial agents. It does not require permanently suppressing and transforming the gut biome or the vaginal flora. There is no risk of fostering antibiotic-resistant organisms and spreading them. 

If treatment for women proves successful, attention might next be returned to applying the therapy to men with chronic bacterial cystitis, with or without accompanying chronic prostatitis.

## 2. Summary

Great strides have already been made. Thanks to ongoing research, we have come a long way. However, the road ahead is proving complex. 

First, we need to overcome the “gold standard” expectation that healthy urine is always “clear and sterile.” Clearly, it is not. Metagenomic analysis reveals the genetic fingerprints of multiple organisms that are not only present within the bladder, but possibly important to maintenance of bladder health. Disruption of a healthy microbiota in the bladder may be a precondition to chronic bladder infection and its sequelae. 

Lactobacilli seem to play an important, protective role in the preservation of bladder well-being, beyond their direct bactericidal activity. Intravesical probiotics may have a restorative effect on the internal environment of the bladder. They may modify the urobiome and induce protective changes within the bladder mucosa. Targeted, personalized, intravesical probiotic therapy may not only have efficacy in the immediate treatment of an ongoing infection, but also provide long-term advantages in the promotion of a healthier intravesical environment, reducing the risk of subsequent infections.

While no easy task lies ahead, events seem to be bringing us to the brink of a major leap forward. Advances in microbiology allow us to isolate lactobacilli with specific antibacterial activity. Genomic interventions may allow the artificial development of “designer probiotics”. [8] Specific strains of *Lactobacillus* acidophilus have already been engineered for the development of biotherapeutics [8].

Capitalizing on this personalized, patient-specific approach will require optimal transitional research. It will entail co-ordination with a high-tech research facility that has the capacity to identify *Lactobacillus* activity against the specific uropathogen in each given case. Industrial advances are improving stabilization for production, maintenance and transport of lactobacilli. Next, formal clinical investigation studies would assess the outcome of individually tailored bladder instillations [20]. Experimentation would also be needed to determine (1) the best dose of the initial probiotic instillation and the volume and makeup of the vehicle, and (2) the need for repeated instillations, immediately or long-term periodically. 

Urine sample kits, available for home collection and direct submission, are now able to expedite the sophisticated NGS identification of the uropathogen specific for a given patient [30,31]. The feasibility and safety of intravesical instillation of lactobacilli has been demonstrated clinically.

Many important pieces seem to be falling into place. The time is right. Together, we may well have an extraordinary opportunity to spearhead a major advance in the prevention of rUTIs in women. Probiotic IntraVesical Organic Therapy (PIVOT) may prove pivotal to this radically new approach. Our goal will be to focus on identifying personalized treatment, specifically targeted for each individual patient. The battle against UTIs can be won, if we concentrate on winning one small skirmish at a time—taking aim with the right weapon (probiotic), at the right target (uropathogen), in the right battlefield (our patient’s bladder). 
